# Extensive Simulated Diving Aggravates Endothelial Dysfunction in Male Pro-atherosclerotic ApoE Knockout Rats

**DOI:** 10.3389/fphys.2020.611208

**Published:** 2020-12-23

**Authors:** Simin Berenji Ardestani, Vladimir V. Matchkov, Kasper Hansen, Nichlas Riise Jespersen, Michael Pedersen, Ingrid Eftedal

**Affiliations:** ^1^MEMBRANES, Department of Biomedicine, Faculty of Health, Aarhus University, Aarhus, Denmark; ^2^Department of Circulation and Medical Imaging, Faculty of Medicine and Health Sciences, NTNU Norwegian University of Science and Technology, Trondheim, Norway; ^3^Comparative Medicine Lab, Department of Clinical Medicine, Aarhus University, Aarhus, Denmark; ^4^Department of Forensic Medicine, Aarhus University, Aarhus, Denmark; ^5^Section for Zoophysiology, Department of Biology, Aarhus University, Aarhus, Denmark; ^6^Department of Cardiology, Aarhus University Hospital, Aarhus, Denmark; ^7^Faculty of Nursing and Health Sciences, Nord University, Bodø, Norway

**Keywords:** endothelial dysfunction, apolipoprotein E (apo E), atherosclerosis, acclimatization, saturation diving

## Abstract

**Introduction:**

The average age of the diving population is rising, and the risk of atherosclerosis and cardiovascular disease in divers are accordingly increasing. It is an open question whether this risk is altered by diving *per se*. In this study, we examined the effect of 7-weeks simulated diving on endothelial function and mitochondrial respiration in atherosclerosis-prone rats.

**Methods:**

Twenty-four male ApoE knockout (KO) rats (9-weeks-old) were fed a Western diet for 8 weeks before 12 rats were exposed to simulated heliox dry-diving in a pressure chamber (600 kPa for 60 min, decompression of 50 kPa/min). The rats were dived twice-weekly for 7 weeks, resulting in a total of 14 dives. The remaining 12 non-diving rats served as controls. Endothelial function of the pulmonary and mesenteric arteries was examined *in vitro* using an isometric myograph. Mitochondrial respiration in cardiac muscle tissues was measured using high-resolution respirometry.

**Results and Conclusion:**

Both ApoE KO diving and non-diving rats showed changes in endothelial function at the end of the intervention, but the extent of these changes was larger in the diving group. Altered nitric oxide signaling was primarily involved in these changes. Mitochondrial respiration was unaltered. In this pro-atherosclerotic rat model of cardiovascular changes, extensive diving appeared to aggravate endothelial dysfunction rather than promote adaptation to oxidative stress.

## Introduction

The average age of the diving population is rising. Currently, one third of US recreational divers are above 50 years of age and carry at least one cardiovascular risk factor such as high cholesterol or hypertension (Denoble et al., 2012; Buzzacott et al., 2018). A recent report of diving-related fatalities ascribes cardiac incidents as an important cause of drowning in 50–69-year-old male divers (Casadesus et al., 2019). Cardiovascular disease is a leading cause of death in the general population worldwide (Roth et al., 2017). It is an open question whether extensive diving increases the cardiovascular disease risk as a result of accumulated pathophysiological stress or may conversely protect cardiovascular function through acclimatization.

Clinically overt cardiovascular disease is usually a consequence of preexisting atherosclerosis. Atherosclerosis is an inflammatory vascular disease with a long asymptomatic phase (Toth, 2008). Endothelial dysfunction in resistant arteries is an early marker of atherosclerosis (Lakatta and Levy, 2003; Castellon and Bogdanova, 2016). Studies of atherosclerotic pathology in rodents have been limited since, unlike humans, they do not naturally develop atherosclerosis (Agoston, 2017). However, Apolipoprotein E knock-out (ApoE KO) animals have emerged as a reliable model to assess the early development of endothelial dysfunction in atherosclerotic disease (Getz and Reardon, 2012; Wei et al., 2015; Rune et al., 2018; Lee et al., 2019). In a previous study, we have shown that male ApoE KO rats develop signs of pro-atherosclerotic changes when fed a high-fat western diet (Berenji Ardestani et al., 2020).

During diving, the cardiovascular system is continuously exposed to oxidative stress due to the production of free radicals and reactive oxygen species (ROS) triggered by factors in the hyperbaric environment (Nossum et al., 1999; Nossum et al., 2002; Brubakk et al., 2005; Eftedal et al., 2012, 2013; Mazur et al., 2014). However, the body’s antioxidant defense system is normally capable of neutralizing the oxidative stress developed during diving (Freitas et al., 2010; Perovic et al., 2014). The idea that repetitive exposure to non-detrimental levels of physiological stress may stimulate protection via adaptation is described in exercise physiology, where regular exercise, depending on the load and basal level, has been shown to increase oxidative stress resistance, angiogenesis, mitochondrial biogenesis and muscle hypertrophy (Gomes et al., 2012). An indication that this may also apply to diving has been reported in the form of persistent increase in the activity of genes involved in apoptosis, inflammation, and innate immune responses in experienced divers (Eftedal et al., 2012).

We have previously shown that a single simulated dive causes endothelial dysfunction in the pulmonary arteries of male ApoE knockout rats (Berenji Ardestani et al., 2019). Endothelial dysfunction in the pulmonary arteries of ApoE KO rats was associated with altered production or bioavailability of NO and was more profound in male than in female rats (Berenji Ardestani et al., 2019). In the present study, we investigated functional changes in the endothelium of pulmonary and mesenteric arteries following extensive diving in the same animal model. We also examined the effect of extensive diving on mitochondrial respiration.

## Materials and Methods

### Ethics Statement

The experiment was conducted in accordance with Animal Research: Reporting of *In Vivo* Experiments (ARRIVE) and the European Convention for the Protection of Vertebrate Animals used for Experimental and Other Scientific Purposes, on permission from the Animal Experimentation Council of the Ministry of Environment and Food of Denmark, approval number 2018-15-0201-01477.

### Animals

Twenty-four male 9-weeks-old (397.0 ± 8.0 g) ApoE KO rats (Horizon Discovery, Saint Louis, United States) were delivered to the animal facility at Aarhus University. Following a 2-week acclimatization period, the rats were fed a Western diet (41% energy from fat, Cat. #D12079B, Brogaarden Korn & Foder ApS, Lynge, Denmark) for a period of 8 weeks, before the diet was changed to standard chow [Special diet service (SDS); Scanbur, Copenhagen, Denmark]. Rats were then randomly divided into diving and non-diving groups at the age of 19-weeks-old. The animals were housed 2 per cage, one diving and one non-diving individual in each cage, with *ad libitum* access to water and food, temperature 21 ± 1°C, and 12–12 h light-dark cycle.

### Simulated Diving Protocol

**FIGURE 1:**
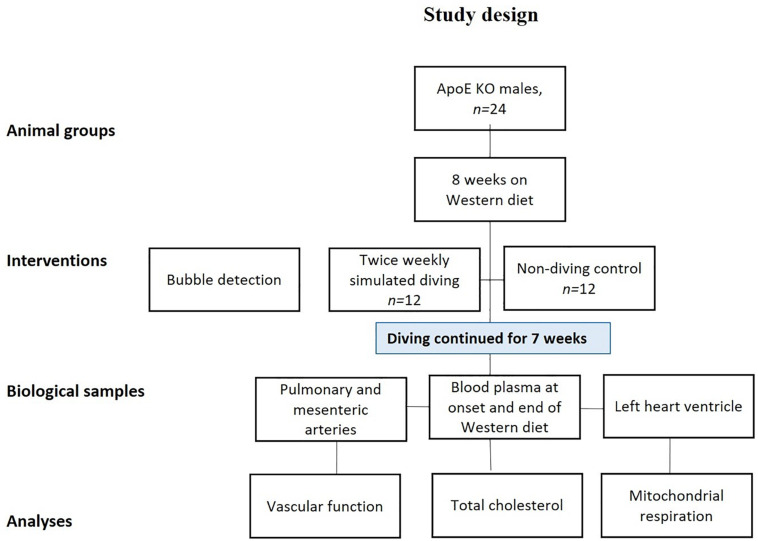
Study design: all rats were fed a Western diet for a period of 8 weeks and were then randomly divided into two groups; a simulated diving and a non-diving control group. The diving group was dived twice/week for 7 weeks. The diving groups were scanned twice for decompression-induced gas bubbles after first and the last (14th) dive. The scanning was performed by using ultrasound system.

Simulated diving was initiated when the rats were 19-weeks-old, using a 60 L hyperbaric chamber, pressurized with a heliox gas mixture (80% He, 20% O_2_). Experiments started at 08:00 in the morning, with six rats exposed simultaneously. During simulated diving the rats were free to move around in the pressure chamber. Compression was 200 kPa/min until 600 kPa, corresponding to 50 meters of seawater (msw), which was maintained for 60 min prior to decompression at a rate of 50 kPa/min (Figure 1). The diving intervention was repeated two times every week (Tuesday and Friday) for a period of 7 weeks, resulting in a total of 14 dives per rat.

### Post-diving Observation for Bubbles and Signs of Decompression Sickness

All diving rats were monitored for decompression-induced inert gas bubbles in the blood immediately after the first and last dive using a preclinical ultrasound system (VisualSonics VEVO2100, Amsterdam, Netherlands). B-mode images of the pulmonary artery were acquired using a 21 MHz transducer (model MS250, VisualSonics). Prior to ultrasound examination, the rat was anesthetized with Sevofloran (100%, Sevorane, AbbVie^®^, Copenhagen, Denmark) evaporated in oxygen, and the hair of the chest was removed using a hair trimmer and hair-removal cream (Veet, Reckitt Benckiser, United States). The rat was placed on the heated imaging platform of the VEVO2100 system while breathing Sevofloran-gas through a facial mask. Heart rate and core temperature (rectal probe) were monitored continuously. The ultrasound examination took approximately 60 min to complete for six rats.

For dives where a subsequent ultrasound examination was not performed, the rats were observed for 15 min after decompression to identify any signs of decompression sickness (DCS) characterized by difficulty breathing, abnormal walking, paralysis and twitching/convulsion (Pontier et al., 2009).

### Blood Sampling

Blood samples were obtained from the tail vein while the rats were under sevoflurane anesthesia at the onset and end of the Western diet. Blood was transferred to 500 μL EDTA tubes (Microvette^®^ 500 K3E, Hounisen, Skanderborg, Denmark) and centrifuged at 4000 *g* for 10 min at 4°C within 30 min of collection. Aspirated plasma was stored in Eppendorf tubes at −80°C until further analysis.

Following 8 weeks of Western diet and 7 weeks of diving, when the rats were 25–28 weeks old, they were anesthetized by subcutaneous injection of a mixture of midazolam (0.5 mg/kg body weight) and fentanyl citrate (0.158 mg/kg body weight) (Jespersen et al., 2017), and euthanized by decapitation. Blood was collected from the left ventricle using vacutainer 4 mL plastic EDTA tubes and stored in −80°C as stated earlier.

### Tissue Dissections

Immediately after euthanasia, the pulmonary artery (first bronchial artery in the right lobe) and third order branches of the mesenteric artery were dissected and transferred to cold (4°C) physiological saline solution (PSS): NaCl, 119 mM; KCl, 4.7 mM; KH_2_PO4, 1.18 mM; MgSO4, 1.17 mM; NaHCO_3_, 25 mM; CaCl_2_, 1.6 mM; EDTA, 0.026 mM; and glucose, 5.5 mM, gassed with 5% CO_2_ in air, and pH adjusted to 7.4.

### Isometric Force Measurement

Each artery was cleaned with dissection scissors and forceps under a microscope, and ∼2 mm lengths of artery were collected and mounted in an isometric wire-myograph (Danish Myo Technology, Aarhus, Denmark). The myograph chamber was filled with PSS and heated to 37°C while constantly bubbled with gas (5% CO_2_ in air). Force (in mN) was recorded with a PowerLab 4SP and Chart5 acquisition system (AD Instruments, Dunedin, New Zealand) and converted to wall tension (in N/m) by dividing the force with twice of the vessel segment length.

Maximal contractile responses were obtained with 119.88 mM K^+^ in the bath solution by using K-PSS (partial substitution of Na^+^ in PSS). Contractility of pulmonary and mesenteric arteries was tested by cumulative applications of the thromboxane analog, U46619 (10^–8^ to 3 × 10^–6^ M), and noradrenaline (NA; 10^–8^ to 3 × 10^–5^ M), respectively. Endothelial function was assessed by relaxing pre-constricted arteries with acetylcholine (ACh: 10^–8^ to 3 × 10^–5^M for mesenteric and 10^–8^ to 3 × 10^–6^ for pulmonary arteries). Pre-constriction to approximately 70% of maximal constriction was obtained with either U46619- or NA-stimulations of the pulmonary and mesenteric arteries, respectively. Different components of endothelium-dependent relaxation were assessed via pre-incubation (20 min) of arteries with various inhibitors including the non-selective NO synthase inhibitor, Nitro-L-arginine methyl-ester (L-NAME;100 μM); the non-selective cyclooxygenase inhibitor, indomethacin (3 μM) and inhibitors of small and intermittent Ca^2+^-activated K^+^ channels, TRAM-34 (1 μM) and apamin (50 nM), which inhibit the endothelium-dependent hyperpolarizing (EDH) response. At the end of each experiment, endothelial-independent relaxation was tested by adding cumulative concentrations of sodium nitroprusside (SNP, 10^–8^–3 × 10^–5^ M). All drugs were purchased from Sigma-Aldrich (Oslo, Norway). No time effect was observed in separate time-control experiments. The protocol was described previously (Berenji Ardestani et al., 2019).

### Mitochondrial Respiration

Mitochondrial respiration was measured by high-resolution respirometry using an oxygraph setup (Oroboros, Innsbruck, Austria) as described elsewhere (Jespersen et al., 2017). Cardiac muscle biopsies from the anterior wall of the left ventricle of 8 randomly selected rats in each group were dissected and stored in BIOPS buffer (BIOPS; in mmol/L: 2.77 CaK_2_ EGTA, 7.23 EGTA, 20 taurine, 6.56 MgCl_2_, 5.77 ATP, 15 phosphocreatine, 0.5 dithiothreitol, and 50 4-morpholineethanesulfonic acid; pH 7.1) at 4°. Cardiac fibers (∼1.5 mg) were permeabilized using saponin and transferred to the oxygraph chamber filled with MiR05 (in mmol/L: 110 sucrose, 60 K-lactobionate, 0.5 EGTA, 0.1% BSA, 3 MgCl_2_, 20 taurine, 10 KH_2_PO_4_, and 20 Hepes; pH 7.1). Substrates and inhibitors were added to the chamber to allow estimation of complex I and Complex I + II linked respiration, and the respiratory control ratio (RCR): (i) complex I (non-ADP-stimulated respiration) tested by glutamate (10 mmol/L) and malate (2 mmol/L); (ii) complex II (ADP-stimulated respiration) tested using glutamate and malate and ADP (5 mmol/L); (iii) mitochondrial outer membrane integrity was tested using cytochorome C (10 μmol/L) complex I + II linked respiration tested using glutamate, malate and succinate (10 mmol/L); (iv) complex I + II linked respiration tested using glutamate, malate and succinate (10 mmol/L); (v) inner mitochondrial membrane integrity was tested with the complex V inhibitor oligomycin (2 μg/mL). Finally, rotenone and antimycin A were used to estimate the residual oxygen consumption. The protocol is summarized in Supplementary Figure S1.

### Total Cholesterol

Total cholesterol was measured in blood plasma using cholesterol-reagents (Randox CH201; Randox Laboratories, Crumlin, United Kingdom). Absorbance was measured at 500 nm at 37°C (PHERAstar; BMG Labtech, Ortenberg, Germany) as described elsewhere (Berenji Ardestani et al., 2019).

### Oil Red O Staining

The aorta was isolated, cleaned from surrounding tissue and fixed in 4% PFA (Paraformaldehyde, Alfa Aesar, United States) at 4°C overnight, and transferred to 1% PFA and stored at 4°C until staining. The aorta was cut and opened through the lower curvature of the aortic arch and the entire length of the vessel. The vessel was placed in Oil Red O (Sigma-Aldrich, Oslo, Norway) working solution at 37°C for 10 min. Then, the vessel was transferred to isopropanol and shortly after (30 s) to sterilized water. The aorta was placed *en face* on the glass and imaged under a microscope.

### Computed Tomography (CT)

Four randomly selected rats in each group were scanned using a clinical available system (Toshiba Aquilion Prime SP): Energy level; 120 kV, tube current; 380 mA, slice thickness; 0.5 mm. Stereological assessment of fat volume was performed using Fiji software FIJI (ImageJ2) (Rueden et al., 2017). In short, a non-destructive grid-overlay of points representing a predefined area was created using the “Multipurpose grid” macro, and the “Cell Counter” plugin of FIJI was then used to mark (count) relevant points overlaying fat tissue in cross sectional (axial) CT images. Analysis was performed after adjusting the viewer to optimal fat visualization (In Hounsfield Units: Window level ∼70, Window width ∼300). Absolute volume was calculated from the number of points using spatial information embedded in the CT-data.

### Data Analysis

Vessel contraction was expressed relative to the maximal contraction induced by KPSS (100% of contraction). Vessel relaxation was expressed as a percentage of the pre-constricted level (0% relaxation) to the passive wall tension (100% relaxation). The effect of inhibitors were calculated as a comparison of difference in concentration-response curves before and after administration of the drug. Concentration–response curves were fitted to experimental data using four-parameter non-linear regression curve fitting. From these curves, –logEC_50_, where EC_50_ is the concentration required to produce a half-maximal response, and maximal contractile (Emax) or maximum relaxation (Rmax) responses were derived and compared using an extra sum-of-squares *F*-test with results presented as means ± SEM (standard error of the mean). Differences between means were tested by one-way ANOVA followed by Bonferroni *post hoc*-test or by *t*-test statistics and the results presented as means ± SD (standard deviation of the mean). Mitochondrial respiration and CT data were analyzed by Student’s *t*-test.

## Results

Two rats from each group (four rats in total) were excluded from the study, either because they died after simulated diving or due to a technical problem with data collection in the non-diving group.

### Body Mass and Total Cholesterol

**FIGURE 2:**
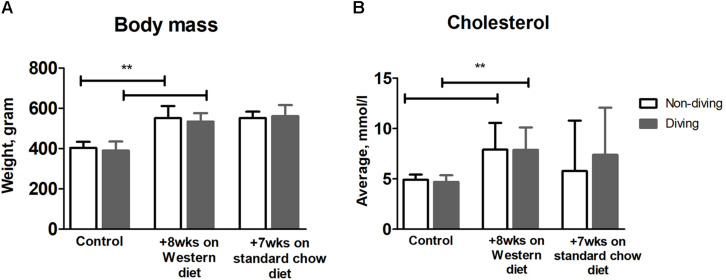
**(A)** Average body mass and **(B)** total cholesterol level over time, in diving vs. non-diving ApoE KO rats after 8 weeks on a Western diet followed by 7 weeks of standard chow diet; **P < 0.0001, ANOVA ± SD, *n* = 12.

There was a significant increase in body mass for all rats after 8 weeks on a Western diet (Figure 2A, *p* < 0.0001); there was no difference in body mass between diving and non-diving groups before or after 7 weeks of simulated diving (Figure 2A, *p* < 0.62). Total cholesterol level displayed a statistically significant increase in both groups after 8 weeks of Western diet (Figure 2B, *p* < 0.0001); there was a decrease in total cholesterol after 7 weeks of standard chow diet in both diving and non-diving group (Figure 2B, *p* < 0.58).

### Ultrasound Imaging and Decompression Stress

After the first dive, one rat died following decompression, and an immediate dissection revealed a large amount of bubbles in the inferior vena cava and heart. After the 7th dive, another rat died, with dissection similarly revealing a large number of bubbles. These rats were not examined with ultrasound. The latter case was caused by human error, as the rat underwent two diving simulations in 1 day. No bubbles were detected in any of the remaining rats using ultrasound (Supplementary Figure S2). Two rats had difficulty breathing after dives 8 and 13, respectively; both recovered within 30 min of observation and were included in all subsequent measurements. No other signs of decompression sickness (DCS) were observed.

### Pulmonary Arteries From Diving Rats Had Reduced NO Production

**FIGURE 3:**
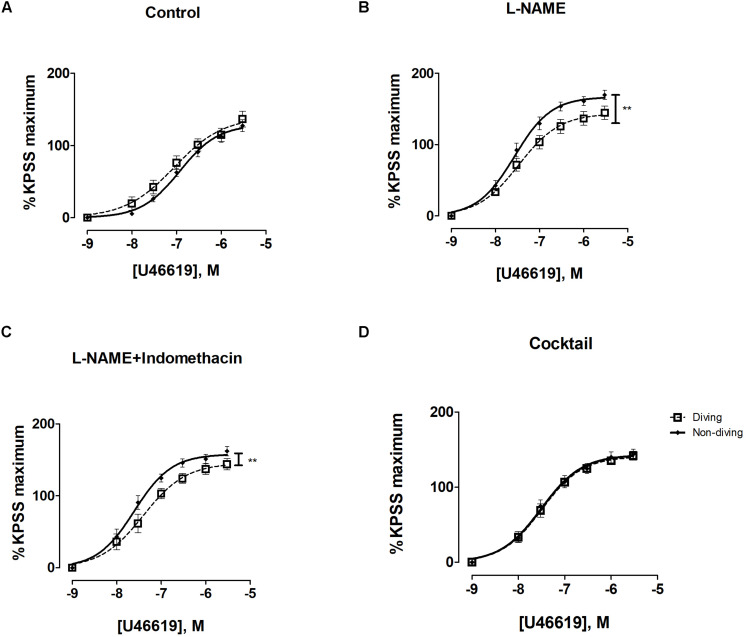
U46619 concentration-response curves of pulmonary arteries from diving and non-diving groups; **(A)** control conditions; **(B)** incubation for 20 min with non-selective inhibitor of NO synthase, L-NAME (100 μM), **p < 0.0001 diving vs. non-diving; **(C)** incubation for 20 min with both L-NAME (100 μM) and indomethacin (3 μM), **p < 0.003 diving vs. non-diving; **(D)** 20 min incubation with cocktail: L-NAME, indomethacin, TRAM-34 and apamin; *F* test ± SE, *n* = 10.

In pulmonary arteries, there were no differences in contractile responses to KPSS between diving and non-diving groups (Supplementary Figure S3). Pulmonary arteries of both diving and non-diving groups contracted in response to U46619 under the control condition, and the diving group tended to be more sensitive to U46619 than the non-diving group (Figure 3A, −logEC50, 7.05 ± 0.16 vs. 6.93 ± 0.08, *p* = 0.06, *n* = 10).

Following 20 min incubation with L-NAME, the contraction of pulmonary arteries to U46619 from both groups was significantly potentiated (Supplementary Figure S4, *P* < 0.0001). The effect of L-NAME was larger in non-diving rats than diving rats. In the presence of L-NAME, pulmonary arteries from non-diving animals were significantly more contractile to U46619 than those from dived rats (Figure 3B; Emax, 143.6 ± 7.14 vs. 166.8 ± 5.49, *P* < 0.0001, respectively; *n* = 10).

A combination of L-NAME and indomethacin did not change the contractile response in the groups (Supplementary Figure S4). Compared to L-NAME alone, L-NAME in combination with indomethacin did not further enhance sensitivity to U46619 (Figure 3C; −logEC50, 7.40 ± 0.10 vs. 7.60 ± 0.06, Emax, 145.5 ± 8.05 vs. 157.9 ± 5.49, *P* < 0.003, respectively; *n* = 10).

After pharmacological inhibition of all major pathways for endothelium-dependent relaxation, i.e., pre-incubation with L-NAME, indomethacin, TRAM-34 and apamin, there was no difference between the non-diving and diving group (Figure 3D; −logEC_50_, 7.49 ± 0.06 vs. 7.505 ± 0.07, respectively; *n* = 10). The contractile response in the diving group was not changed but the non-diving group response was significantly suppressed (Supplementary Figure S4, *p* = 0.001).

**FIGURE 4:**
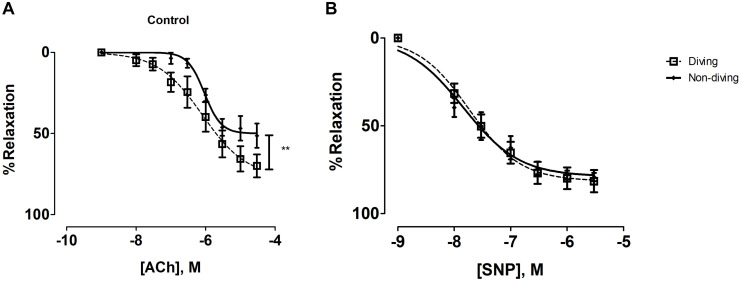
**(A)** Concentration-response curves of pulmonary arteries from diving and non-diving ApoE KO rats to ACh under control conditions, **P < 0.003; **(B)** Concentration-response curves of pulmonary arteries to SNP; *F*-test, *n* = 10.

There was a statistically significant difference between the diving and non-diving groups in ACh-induced relaxation responses; arteries from the diving group showed more ACh-induced relaxation compared to arteries from the non-diving group (Figure 4A; Rmax, 76.99 ± 12.39 vs. 49.97 ± 3.96, *P* < 0.003, respectively; *n* = 10).

In the presence of L-NAME, relaxation was abolished in both groups and all pulmonary arteries constricted in response to ACh. This contractile response was greater in the diving group compared to the non-diving group (Supplementary Figure S5; −logEC_50_, 8.01 ± 0.53 vs. 7.78 ± 0.52, respectively; *n* = 10).

Incubation with indomethacin and L-NAME alone, or with L-NAME, indomethacin, TRAM-34 and apamin, both abolished the relaxation responses to ACh in the diving- and non-diving groups (Supplementary Figure S5).

Endothelial independent relaxation was tested by SNP. No difference between the diving- and non-diving groups was observed (Figure 4B).

### Mesenteric Arteries From Diving Rats Had Reduced NO Production

**FIGURE 5:**
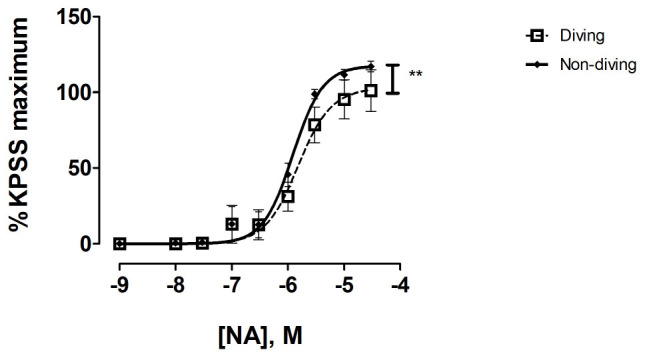
Concentration response curve of mesenteric arteries from diving and non-diving ApoE KO rats to noradrenaline; *F*-test ± SE, *n* = 10, **p < 0.001.

There were no differences between the contractile response to KPSS in pulmonary arteries from diving and non-diving rats (Supplementary Figure S3). There was a significant difference in contractile response of mesentery arteries, where the non-diving group constricted stronger to NA relative to the dived group (Figure 5; Emax, 117.4 ± 4.63 vs. 102.7 ± 9.63, *P* = 0.03, respectively; *n* = 10).

**FIGURE 6:**
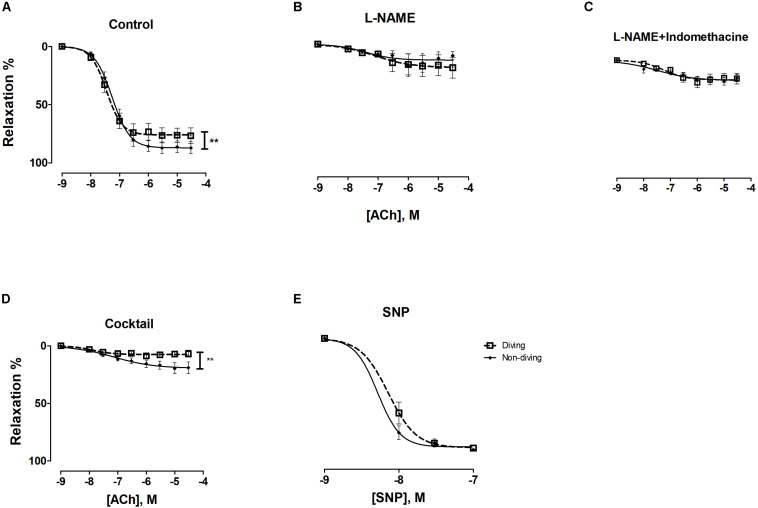
ACh concentration-response curves of mesenteric arteries from diving and non-diving groups; **(A)** control conditions, **P = 0.01; **(B)** 20 min incubation with non-selective inhibitor of NO synthase, L-NAME (100 μM); **(C)** 20 min incubation with L-NAME (100 μM) and indomethacin (3 μM); **(D)** 20 min incubation with cocktail: L-NAME, indomethacin, TRAM-34 and apamin, i.e., pharmacological inhibition of all major pathways for endothelium-dependent relaxation, **p < 0.0002; **(E)** SNP; *F*-test ± SE, *n* = 10.

ACh-induced relaxation responses of pre-constricted mesentery arteries were also significantly different between the diving and non-diving group. The arteries from non-diving rats showed more ACh-induced relaxation than the arteries from diving rats (Figure 6A; Rmax, 787.0 ± 2.29 vs. 76.0 ± 2.81, *P* = 0.008, respectively; *n* = 10).

Incubation with L-NAME significantly suppressed the relaxation responses to ACh in both groups (Supplementary Figure S6). The differences in ACh concentration-response curves between non-diving and diving rats disappeared in the presence of L-NAME (Figure 6B; −logEC_50_, 7.08 ± 0.60 vs. 7.49 ± 0.52, respectively; *n* = 10), suggesting an increased NO pathway contribution to the ACh response in the non-diving compared to diving group.

Incubation with both L-NAME and indomethacin caused no further inhibition of the ACh-induced relaxation than L-NAME alone (Supplementary Figure S6 and Figure 6C).

Incubation with TRAM-34 and apamin further suppressed relaxation of mesentery arteries from diving rats (Supplementary Figure S6, *p* < 0.001) than incubation with L-NAME and indomethacin. There was a significant difference between the diving- and non-diving group (Figure 6D; Rmax, 7.46 ± 1.03 vs. 19.44 ± 2.83, *p* = 0.004, respectively; *n* = 10). The relaxation responses to SNP were similar in both groups (Figure 6E).

### Mitochondrial Respiration Was Unaltered

**FIGURE 7:**
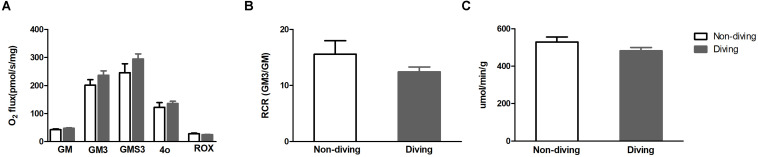
**(A)** Mitochondrial respiration capacity after addition of substrates; GM (state 2), glutamate and malate; GM3 (state 3) glutamate and malate; GMS3 (state 3), glutamate, malate and succinate; 4o (state 4), oligomycin; ROX, residual oxygen consumption **(B)** respiratory control ratio (RCR); **(C)** Citrate synthase (an index for mitochondrial numbers), *t-test* ± SD, *n* = 8.

There was no difference in mitochondrial respiration capacity between diving and non-diving rats. When tested alone or in combination, the activity of complex I + II was similar in both groups at the end of simulated diving intervention (Figure 7A). The RCR (Figure 7B) and oxygen consumption unrelated to respiration was also compared between the groups and no difference was found (Figure 7A). We found no differences in citrate synthase activity between the groups (Figure 7C).

### Oil Red O Staining

No difference between diving and non-diving was observed. The magnitude of the ORO-stained lipid areas were too low to quantify in either group (Supplementary Figure S7).

### CT

No difference in fat volume between diving and non-diving was observed (21.08 ± 1.51 vs 22.33 ± 3.15%, respectively).

## Discussion

To our knowledge, the present study is the first to measure the effects of extensive simulated diving on endothelial function and mitochondrial respiration in blood vessels and cardiac tissues from male rats prone to atherosclerosis development. Our main finding was that both diving and non-diving rats showed pro-atherosclerotic endothelial changes in their pulmonary arteries. 7 weeks of simulated heliox diving (resulting in 14 h of ∼126 kPa PO_2_ exposure) further aggravated these pro-atherosclerotic endothelial changes of the pulmonary arteries in the diving group animals. We also observed impairment of endothelial function in mesenteric arteries following diving. Mitochondrial respiration in cardiac tissue was not affected by the simulated diving intervention.

ApoE KOs animal models are widely used to study experimental atherosclerosis. These animals suffer dyslipidemia and vascular inflammation related to excess oxidative stress (Getz and Reardon, 2012; Rune et al., 2018). We showed that male ApoE KOs on a high-fat Western diet developed endothelial dysfunction via changes in COX-dependent relaxation and increased production of endothelium-derived contractile factors (Berenji Ardestani et al., 2020). Diving causes endothelial dysfunction in humans and experimental animals, independently of other pathology such as DCS (Brubakk et al., 2005; Lambrechts et al., 2013). We recently showed that male ApoE KO rats as young as 6–9-weeks-old have greater endothelial dysfunction in their pulmonary arteries than Sprague Dawley rats after a similar simulated dive protocol (absolute pressure of 600 kPa and decompression of 50 kPa/min), due to altered NO-dependent signaling (Berenji Ardestani et al., 2019).

In the present study, when pre-constricted pulmonary arteries were compared under control conditions for their ACh-induced relaxation responses, arteries of diving ApoE KO rats relaxed significantly stronger than those from non-diving controls. However, pulmonary arteries showed tachyphylaxis, as reported earlier, when cumulatively stimulated with increasing concentrations of ACh (Berenji Ardestani et al., 2019). It is therefore difficult to make a clear conclusion from the ACh-induced responses. Instead, contractile responses to U46619 were stable and the non-diving group had a larger contractile response after incubation with L-NAME. This finding suggests a stronger contribution of NO in pulmonary arteries of the non-diving group compared to the diving group; because SNP-induced relaxation was similar in the two groups, which again suggest a similar sensitivity of smooth muscle cells to NO. This is, however, in contrast to our finding with ACh-induced relaxation. The reason for this is unclear but might be due to different states of endothelial activation; in U46619-dependent constriction, where endothelium activity is at a basal level, and during ACh induced contraction, when the endothelium is gradually activated to the maximal level. Thus, one can speculate that the diving group is characterized by lower basal endothelial tone but has lager capacity – primarily via NO – for further activation.

Previous studies have showed that atherosclerosis is accompanied with oxidative stress and increased production of endothelium-derived contractile factors (Gluais et al., 2005; Félétou et al., 2011). NO-mediated endothelium-dependent relaxation was earlier observed to be impaired in ApoE KO mice fed on a Western diet, while there was an upregulation of COX-1 and/or the induction of COX-2 (Yang et al., 2002; Herrera et al., 2010). NO is the most important vasodilator and the elevated amount of O_2_^–^ and oxidative stress has a significant role in deactivation of NO via peroxynitrite formation (van der Loo et al., 2000; d’Uscio et al., 2001; Herrera et al., 2010). This might also be the case at the basal endothelial activity state in our rats. A previous study on ApoE KO rats also showed changes in COX-dependent signaling and increased endothelium-dependent contraction in pulmonary arteries of ApoE KO rats (Berenji Ardestani et al., 2020). In this study, incubation of pulmonary arteries with indomethacin and L-NAME made no difference in neither contractile or relaxation responses between the two groups. The difference in the present result, compared to the previous (Berenji Ardestani et al., 2020), could be due to the lower extent of hypercholesteremia in this current study; rats were fed a Western diet for a period of 8 weeks and total plasma cholesterol level was much lower (7.8 mmol/L) compared to the previous study (36.43 mmol/L). Accordingly, it is possible that the extent of oxidative stress was less (Berenji Ardestani et al., 2019).

Further incubation with EDH signaling inhibitors suppressed the U46619 contractile responses and contraction became similar between the groups. The reason for this effect is unclear but it might be due to previously reported inhibitory action of 1 μM TRAM-34 on non-selective cation channels that contribute to U46619 contractile response (Schilling and Eder, 2007). It is possible that the contribution of these cation channels is less in the non-diving group compared to the dived group. Another option is that the non-selective cation channels in the non-diving group could be balanced by the increased small-conductance or intermediate-conductance calcium-activated potassium channels (SK_*C*_a/IK_*Ca*_) dependent relaxation, as observed in the ACh response. Although we believe the last possibility is most probable, a clear answer will await further investigation. A similar result was reported earlier in mesenteric arteries of ApoE KO rats on a Western diet (Berenji Ardestani et al., 2020). ACh-induced responses in the presence of different endothelium-dependent relaxation blockers in mesenteric artery were similar to those found in pulmonary arteries (Supplementary Figures S5, S6), confirming the elevated contribution of NO in endothelium-dependent relaxation in the non-diving group.

The second aim of this study was to investigate the effect of extensive diving on mitochondrial respiration. Myocardial cells have high mitochondrial density in order to meet the high-energy-demand of cardiac muscle cells (Hall et al., 2014). Earlier studies have shown that mitochondrial dysfunction is involved in the initiation and progression of atherosclerosis (Siasos et al., 2018). Oxidative stress leads to oxidative modification of mitochondrial proteins and ultimately mitochondrial dysfunction (Siasos et al., 2018). On the other hand, there is evidence that both endurance- and resistance training have the capacity to protect against mitochondrial dysfunction and systemic inflammation, which are central hallmarks of atherosclerosis (Groennebaek and Vissing, 2017; Nilsson and Tarnopolsky, 2019).

The molecular mechanisms of mitochondrial adaptation to exercise are debated; current evidence points to the involvement of alterations in intramuscular homeostasis and ROS, and ultimately improved mitochondrial respiration via reduction in mitochondrial oxidant production and enhanced mitochondrial antioxidant enzyme activity (Kavazis et al., 2008).

Reactive oxygen species generation triggered by hyperoxia has been reported to cause mitochondrial depolarization *in vitro* following simulated dives in bovine arterial endothelial cells (Wang et al., 2015). Endogenous ROS is mainly produced by mitochondrial complex I and II. Ischemic reperfusion injuries have been found to increase ROS production from complex I and cause impaired mitochondrial respiration in male Wistar rats (Jespersen et al., 2017). Further, remote ischemic conditioning following simulated diving has been shown to aggravate DCS and even mortality in female Wistar-rats (Schmidt et al., 2016). At normal physiological concentrations, ROS are essential intracellular signaling substances and recognized as important mechanisms in mitochondrial adaption to exercise (Gomes et al., 2012; Jespersen et al., 2017). In the present study, we found no changes in mitochondria respiration capacity in pro-atherosclerotic rats after extensive simulated diving, however there was no attenuation either. We therefore suggest that extensive diving on pro-atherosclerosis rats did not deteriorate the mitochondrial function.

Since this study was not designed to study DCS we deliberately chose to employ a diving protocol that would limits decompression-induced bubble formation. The heliox mixture breathing gas used in this study is recognized as a safer choice than air because the removal of nitrogen from the breathing gas in heliox reduces bubbling (Brubakk et al., 2014). In the present study, two rats died after decompression with massive bubble accumulations in the heart and inferior vena cava; the first died after the initial diving intervention, and the second dead rat was found after the 7th dive. The 2nd rat’s death was due to a human error as the rats was dived twice in 1 day. No other rats had visible DCS symptoms and no bubbles were seen in ultrasound images after the last diving session. In order to limit oxygen toxicity and gas bubble formation in divers, different breathing gas mixtures are recommended, depending on the dive profile (Brubakk et al., 2014). However, large individual and intra-individual variability exists for tolerance to oxygen toxicity, bubble production and DCS risk; the same dive protocol can cause DCS in some individuals and no symptoms in others (Bakovic et al., 2008; Lautridou et al., 2017). Body mass and adipose tissue are associated with DCS risk in both human and rats (Carturan et al., 2002; Buzzacott et al., 2016; Cialoni et al., 2017), and ApoE KO rats have higher body mass and plasma cholesterol levels than Sprague Dawley rats of similar age (Berenji Ardestani et al., 2020). However, since all other rats survived the diving interventions without any observed bubbles or DCS, it is unlikely that hypercholesteremia or heavy body mass were the reason for the significant amount of bubbles in the dead rat.

## Conclusion

In this study we found that 7 weeks of extensive simulated heliox diving worsened endothelial abnormalities in the pulmonary and mesenteric arteries of male ApoE KO rats. These results enhance our understanding about effects of oxidative stress in divers suffering from impaired endothelial function; our results indicate that extensive diving does not protect pro-atherosclerotic arteries in a rat model of endothelial dysfunction.

## Data Availability Statement

The original contributions presented in the study are included in the article/Supplementary Material, further inquiries can be directed to the corresponding author/s.

## Ethics Statement

The animal study was reviewed and approved by the Animal Experimentation Council of the Ministry of Environment and Food of Denmark.

## Author Contributions

SB, VM, and IE designed the study. SB managed the data collection and laboratory work and analysis. SB and VM conducted the statistical analysis. SB drafted the manuscript. All the authors contributed actively in writing and approved the final version.

## Conflict of Interest

The authors declare that the research was conducted in the absence of any commercial or financial relationships that could be construed as a potential conflict of interest.
