# Supplementary figures and images for: Extensive Simulated Diving Aggravates Endothelial Dysfunction in Male Pro-atherosclerotic ApoE Knockout Rats
Source: Front Physiol. 2020 Dec 23;11:611208. doi: 10.3389/fphys.2020.611208 (PMC7786538; doi:10.3389/fphys.2020.611208)

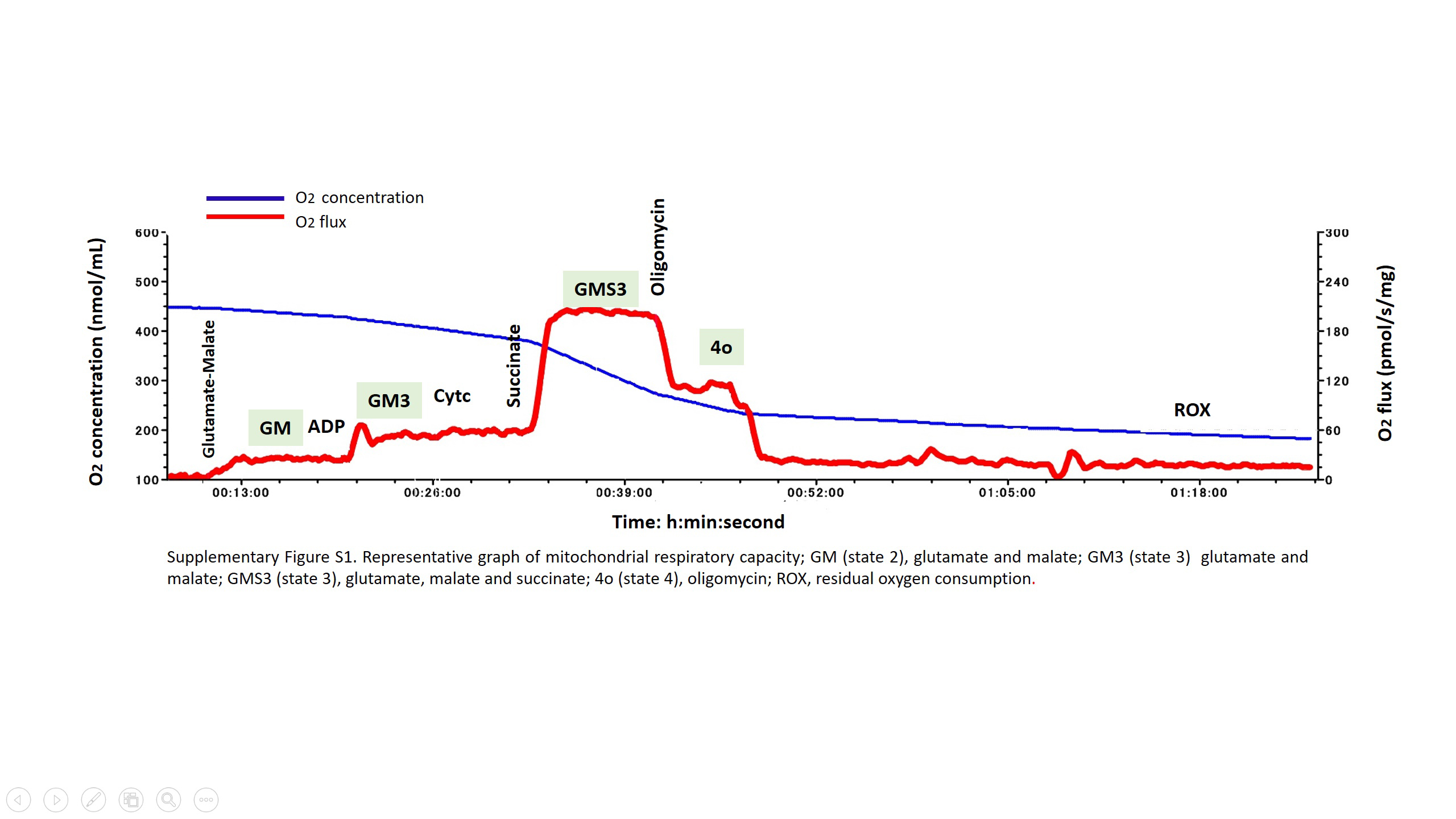

Supplement: Supplementary file 1 [file Image_1.JPEG]

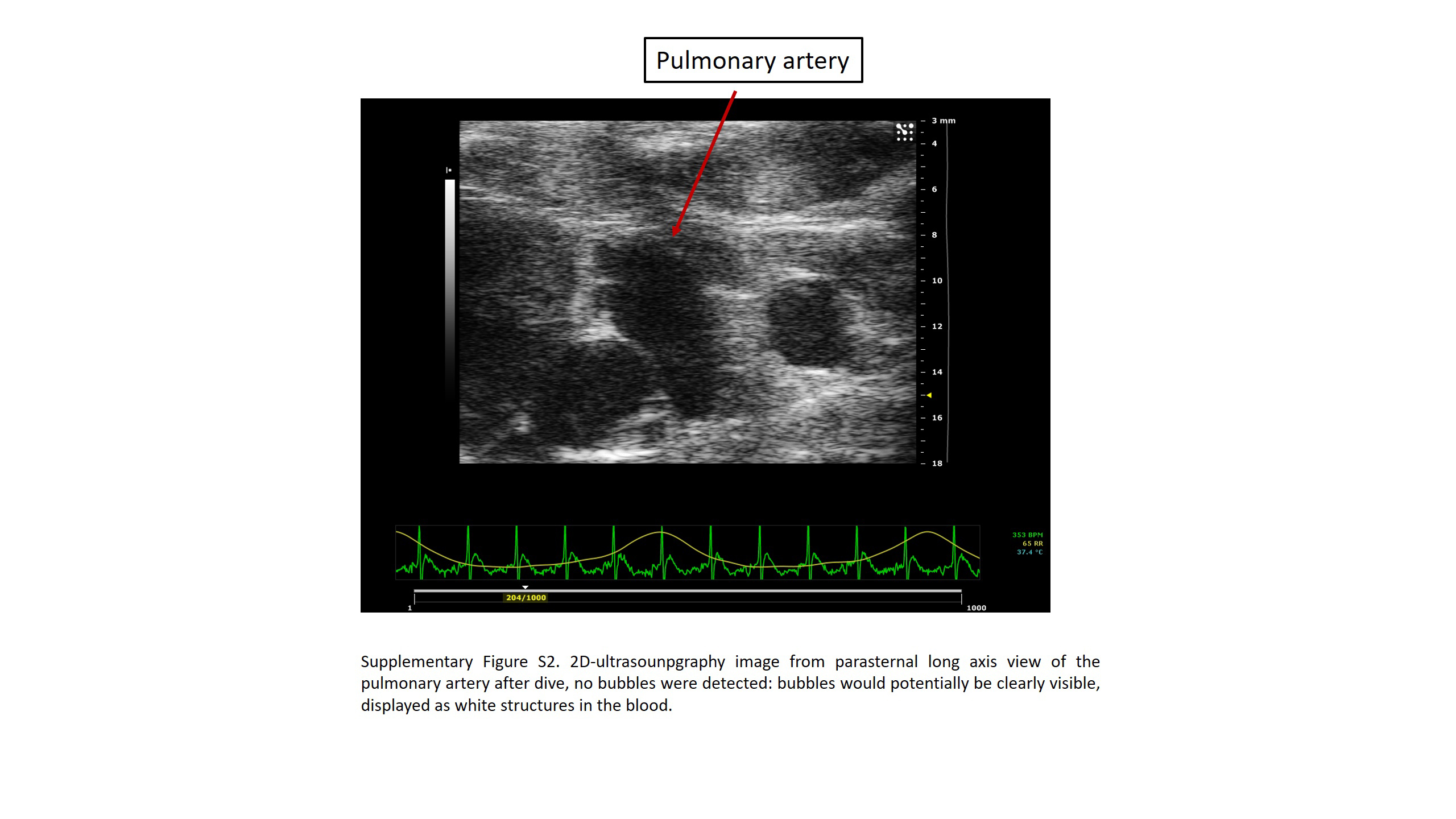

Supplement: Supplementary file 2 [file Image_2.JPEG]

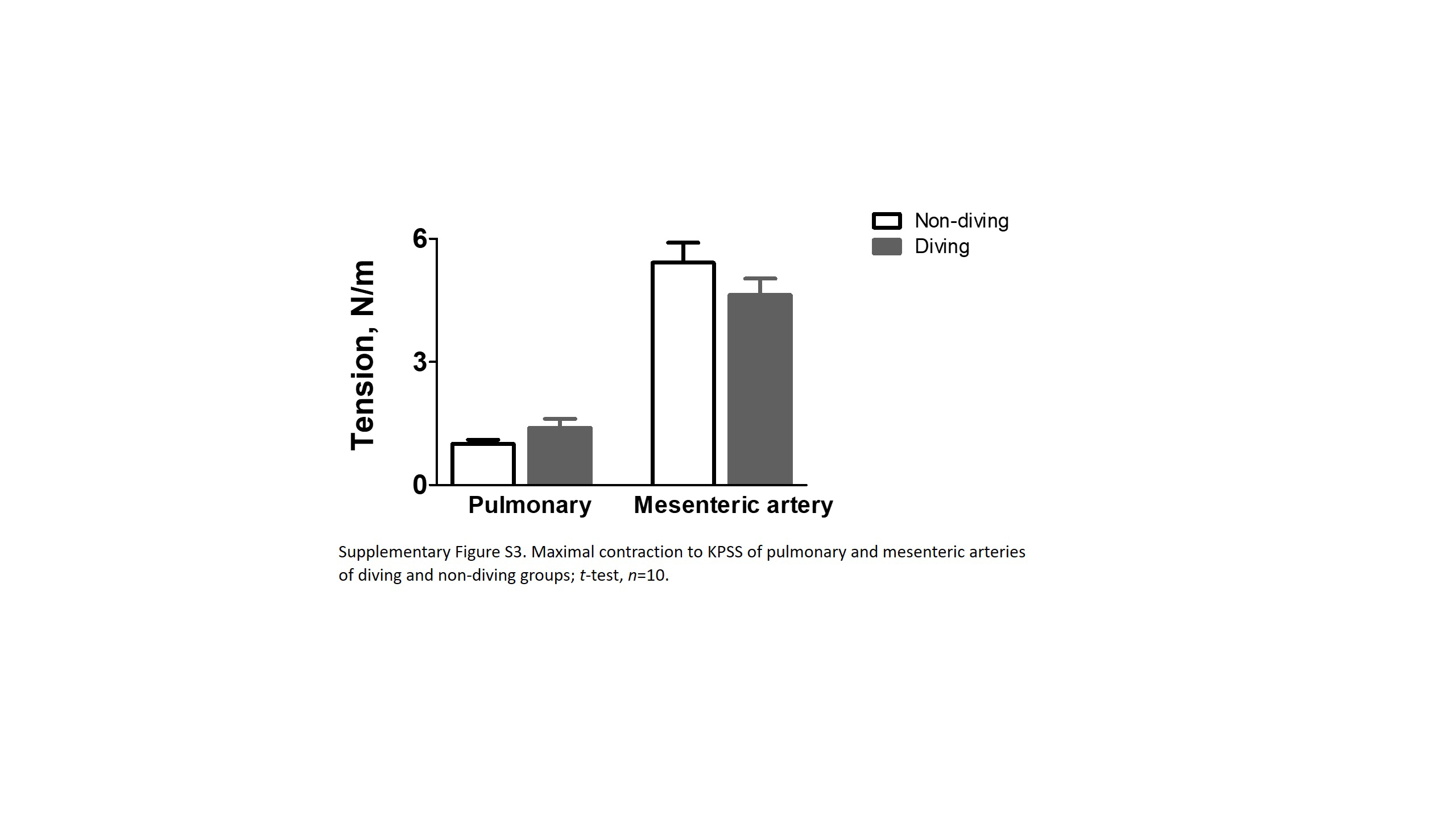

Supplement: Supplementary file 3 [file Image_3.JPEG]

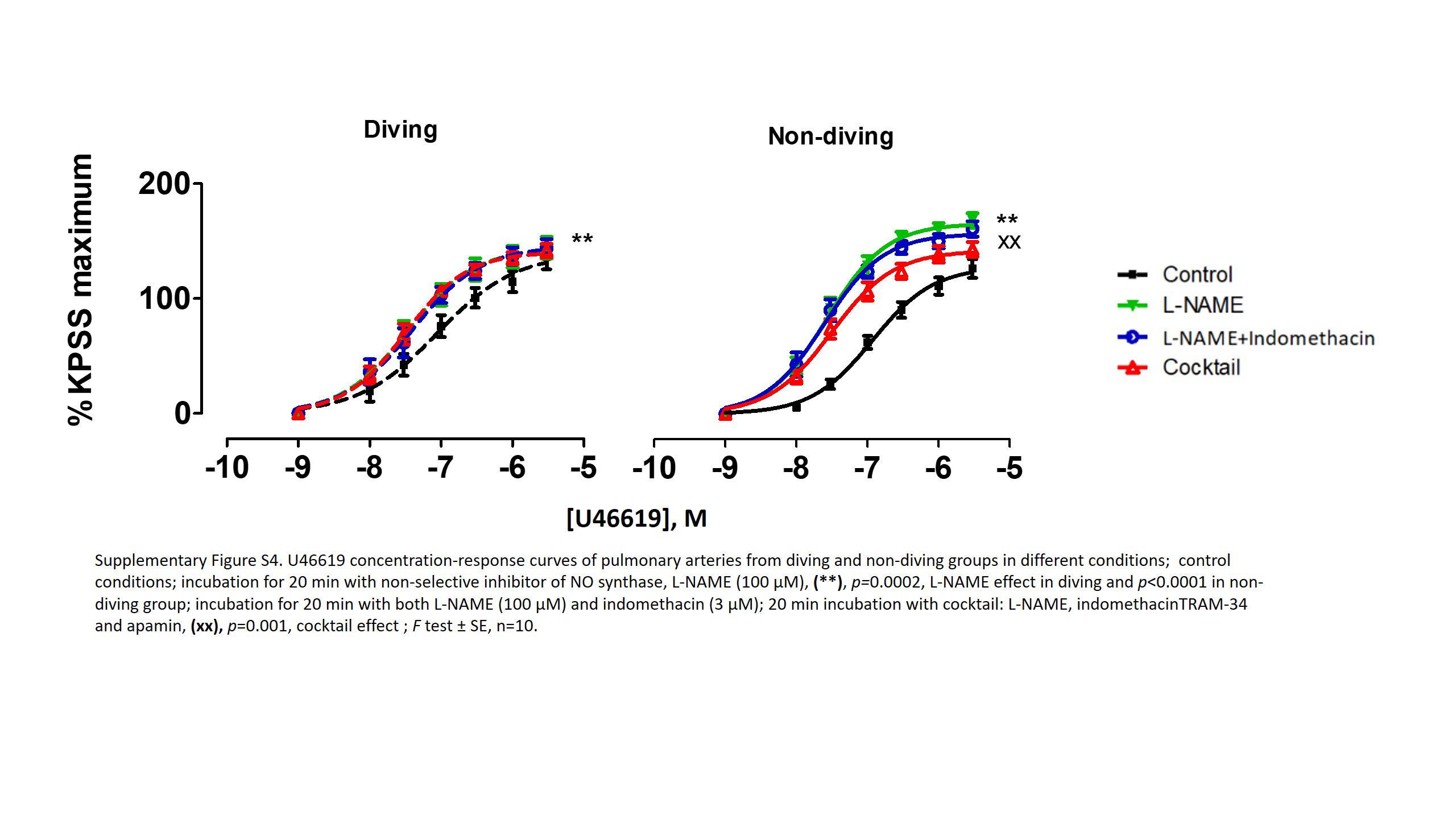

Supplement: Supplementary file 4 [file Image_4.JPEG]

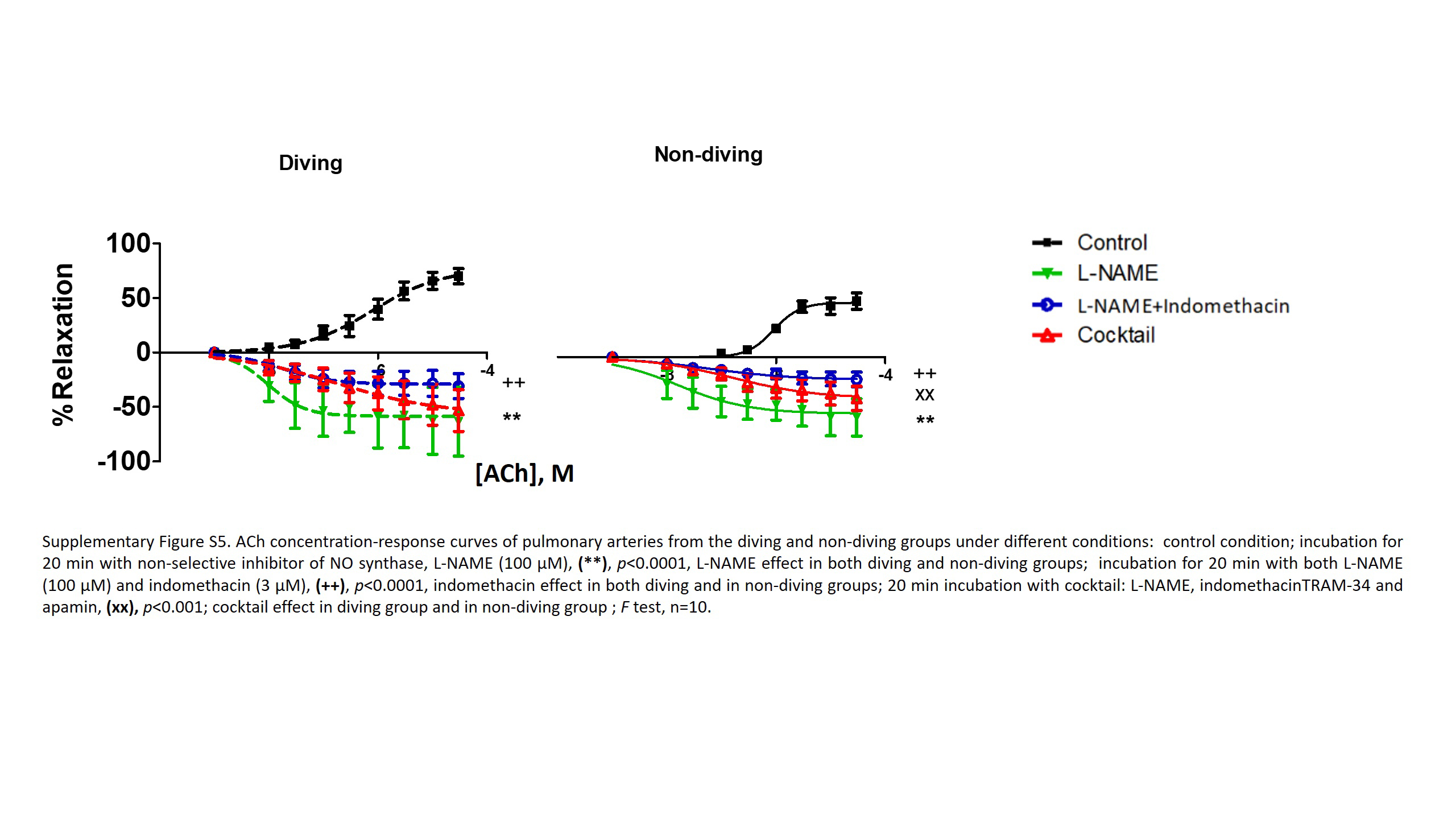

Supplement: Supplementary file 5 [file Image_5.JPEG]

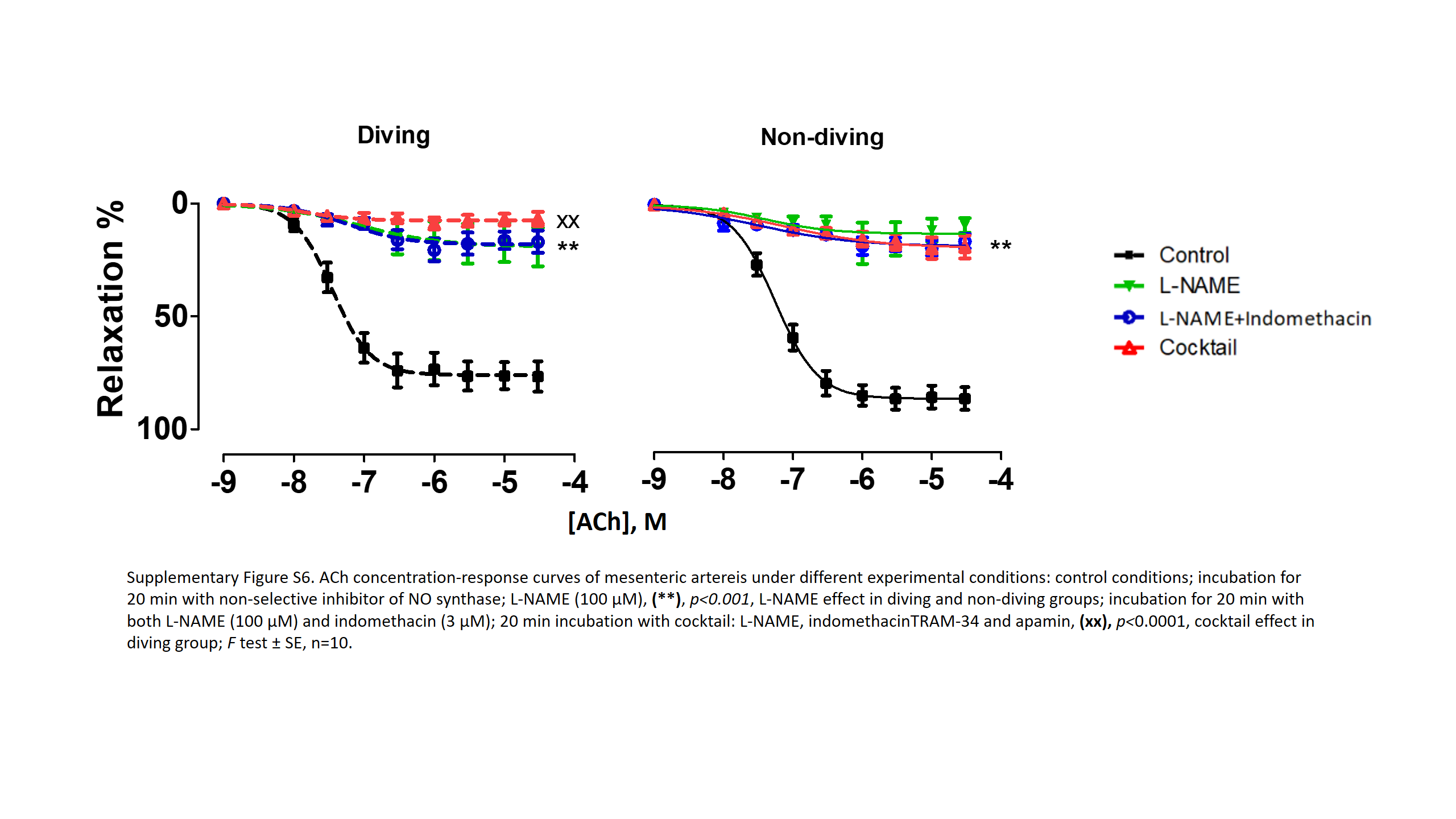

Supplement: Supplementary file 6 [file Image_6.png]

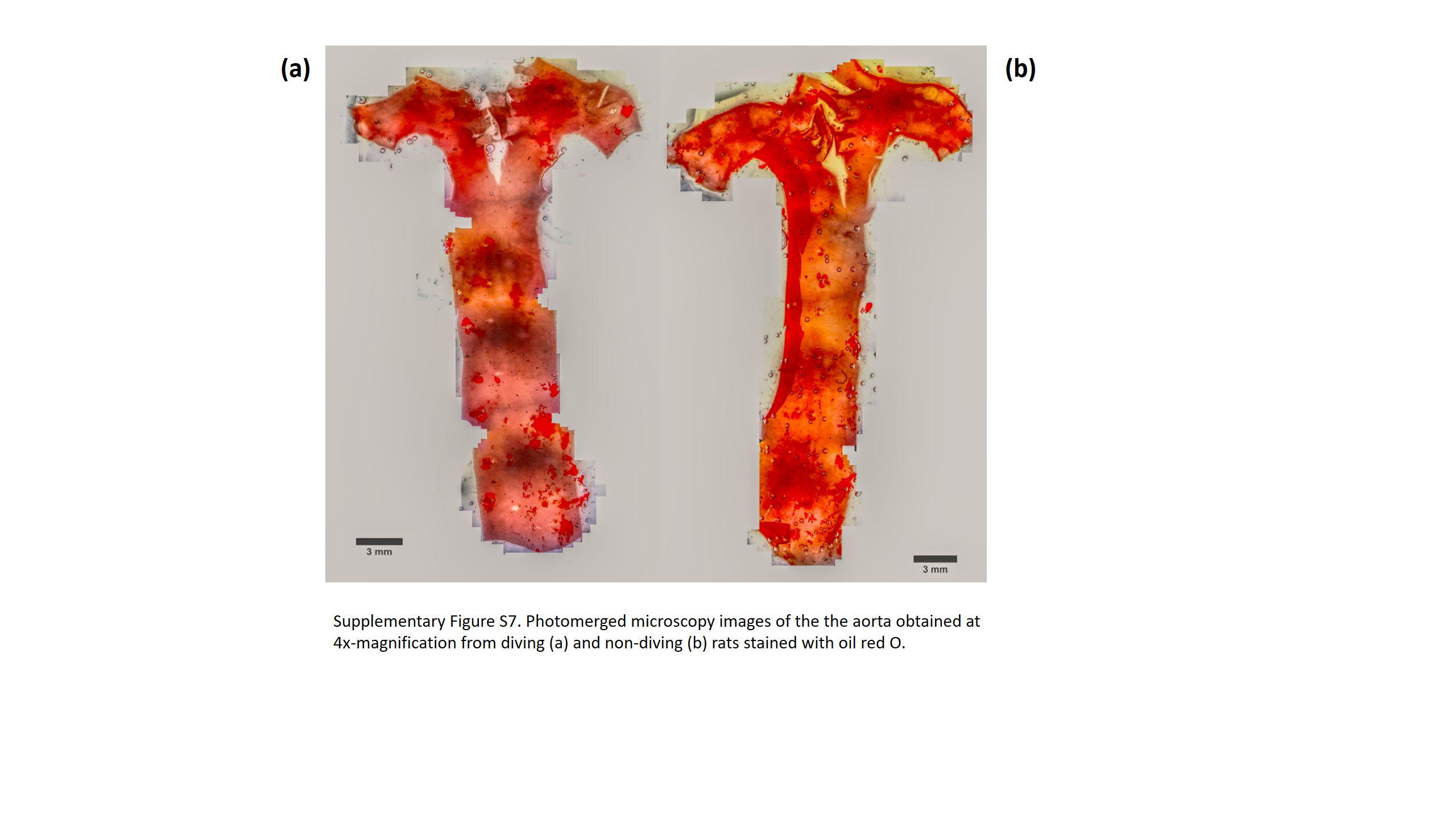

Supplement: Supplementary file 7 [file Image_7.png]
